# Integrated bioinformatic analysis reveals immune molecular markers and potential drugs for diabetic cardiomyopathy

**DOI:** 10.3389/fendo.2022.933635

**Published:** 2022-08-15

**Authors:** Qixin Guo, Qingqing Zhu, Ting Zhang, Qiang Qu, Iokfai Cheang, Shengen Liao, Mengli Chen, Xu Zhu, Mengsha Shi, Xinli Li

**Affiliations:** Department of Cardiology, The First Affiliated Hospital of Nanjing Medical University, Nanjing, China

**Keywords:** diabetic cardiomyopathy, bioinformatics, molecular docking, potential drugs, diabetes mellitus, biomarker

## Abstract

Diabetic cardiomyopathy (DCM) is a pathophysiological condition induced by diabetes mellitus that often causes heart failure (HF). However, their mechanistic relationships remain unclear. This study aimed to identify immune gene signatures and molecular mechanisms of DCM. Microarray data from the Gene Expression Omnibus (GEO) database from patients with DCM were subjected to weighted gene co-expression network analysis (WGCNA) identify co-expression modules. Core expression modules were intersected with the immune gene database. We analyzed and mapped protein-protein interaction (PPI) networks using the STRING database and MCODE and filtering out 17 hub genes using cytoHubba software. Finally, potential transcriptional regulatory factors and therapeutic drugs were identified and molecular docking between gene targets and small molecules was performed. We identified five potential immune biomarkers: proteosome subunit beta type-8 (*PSMB8*), nuclear factor kappa B1 (*NFKB1*), albumin (*ALB*), endothelin 1 (*EDN1*), and estrogen receptor 1 (*ESR1*). Their expression levels in animal models were consistent with the changes observed in the datasets. *EDN1* showed significant differences in expression in both the dataset and the validation model by real-time quantitative PCR (qPCR) and Western blotting(WB). Subsequently, we confirmed that the potential transcription factors upstream of *EDN1* were PRDM5 and KLF4, as its expression was positively correlated with the expression of the two transcription factors. To repurpose known therapeutic drugs, a connectivity map (CMap) database was retrieved, and nine candidate compounds were identified. Finally, molecular docking simulations of the proteins encoded by the five genes with small-molecule drugs were performed. Our data suggest that *EDN1* may play a key role in the development of DCM and is a potential DCM biomarker.

## Introduction

Diabetic cardiomyopathy (DCM) is defined as myocardial dysfunction that develops from complex pathophysiological mechanisms of diabetes in the absence of coronary artery disease (CAD) or hypertension (1). Currently, epidemiological studies have indicated a high incidence (19–26%) of heart failure (HF) in patients with diabetes mellitus (2), creating a heavy financial burden on both government and citizens (3).

However, due to the lack of early symptoms and signs, DCM was often ignored (4). Even in asymptomatic patients with well-controlled diabetes, up to 50% have been found to exhibit some degree of cardiac dysfunction (5). The current treatments for DCM have been traditional cardiac and anti-glycemic drugs, with no targeted therapies (6). Considering that there are no established diagnostic criteria and targeted therapeutics for DCM, the development of diagnostic biomarkers and the identification of therapeutic targets based on its molecular mechanism are of great clinical significance.

Multiple factors contribute to the pathophysiology of DCM, including systemic metabolic disorders, inappropriate activation of the renin-angiotensin-aldosterone system (RAAS), subcellular abnormalities, oxidative stress, inflammation, and dysfunctional immunomodulation (7). With immunological mechanisms contributing to the pathogenesis of HF, immunology has become a significant research topic. The Canakinumab Anti-inflammatory Thrombosis Outcome Study (CANTOS) trial has shown that inflammation is one of the main drivers of atherosclerosis (8). Further exploration of immunology was expected to expand our understanding of the fundamental mechanisms of DCM.

Numerous groups have performed comprehensive, microarray-based, genome-wide analyses. The use of bioinformatics methods and tools to identify disease signatures and drug targets is rapidly maturing. Despite these analyses, addressing DCM by immune-informatics approaches remains largely unexplored. In this study, we integrated bioinformatic analysis to identify immune molecular biomarkers and potential drugs for diabetic cardiomyopathy, further testing them using molecular docking and animal model validation.

## Materials and methods

### Dataset and differentially expressed genes (DEGs)

Raw microarray data from the dataset GSE4745, based on the platform GPL85, were downloaded from the GEO database. These data were converted to gene names and normalized, followed by removal of bias and variability from the dataset using the combat function in the surrogate variable analysis (sva) package. The analysis of digital gene expression using the R (edgeR) package was used to screen for DEGs between diabetic patients and healthy controls. The selection criteria were |log_2_ FC| > 1 and false discovery rate (FDR) < 0.05.

### Immune infiltration analysis

The degrees of infiltration by 22 immune cell types were quantified by transcriptomic data using the CIBERSORT deconvolution algorithm. The cell types investigated included plasma cells, resting memory CD4+ T cells, CD8+ T cells, naïve CD4+ T cells, T follicular helper cells, regulatory T cells (Tregs), activated memory CD4+ T cells, gamma delta T cells, naïve B cells, memory B cells, monocytes, M0 macrophages, M1 macrophages, M2 macrophages, resting natural killer (NK) cells, activated NK cells, activated mast cells, eosinophils, neutrophils, resting dendritic cells, activated dendritic cells, and resting mast cells. The CIBERSORT data were visualized using R software.

### WGCNA and ImmPort database of immune-related genes

WGCNA is an algorithm that finds co-expressed gene modules of high biological significance and explores the relationship between gene networks and diseases. We obtained approximately 5000 genes for further analysis based on P values < 0.05 and logFC > 0.05. The main processes were: (i) the hierarchical clustering (hclust) function was used for hclustanalysis; (ii) the Topological Overlap Matrix (TOM) was constructed using the pickSoftThreshold function to determine the optimal soft threshold; (iii) the expression profile of each module was summarized by module signature genes (ME); (iv) the relationship between ME and DCM was calculated using Pearson correlation analysis; (v) Gene Ontology (GO) annotation and KEGG pathway enrichment analysis were performed for these functional modules and (vi) genes from the immune database intersected with genes from the module.

### Establishment of PPI network topology algorithm and hub node detection

PPIs are a target of cell biology research and a prerequisite for systems biology studies (9). Proteins were placed in PPI networks through interactions with another protein, suggesting its possible function(s). We constructed PPI networks from DEGs using the STRING (https://string-db.org/) repository to describe functional and physical interactions in DCM (10). Hub nodes were generally defined by highly interconnected nodes in large and complex PPI networks. Hub nodes were determined by a degree topology algorithm using cytoHubba (http://apps.cytoscape.org/apps/cytohubba), a Cytoscape plug-in for Cytoscape software (11). PPI network was then analyzed using the MCODE plug-in with default parameters degree cutoff ≥2, node score cutoff ≥2, K-core ≥2 and max depth =100); finally, cytoHubba was used to identify hub genes.

### Gene set enrichment analysis (GSEA) and potential transcription factors (TFs)

Gene sets with enrichment scores with a normalized P < 0.05, and an FDR of < 0.25, were considered significantly enriched. The University of California Santa Cruz (UCSC) sequence database was used to identify promoter sequences of target genes, followed by a search for potential TFs in the JASPAR database, a first screening step based on the direction and correlation level of transcription, and a second step using expression in the GEPIA database and clarification of its binding structural domain.

### Potential therapeutic chemicals and their molecular docking to proteins

Genes of interest were imported into the clue.io platform to identify potentially useful small molecules (**Supplementary Figure 1**). There were three steps for molecular docking. First, the drug candidates were imported into the PubChem database to obtain their three-dimensional structures and minimize their energy using viaChem 3D. Second, target genes were imported into the Universal Protein Resource (UniProt) and Protein Databank (PDB) databases to obtain the highest-resolution receptor structures and perform dehydration, hydrogenation, and charge setting operations (using viaAutoDockTools and PyMOL software). Third, molecular linkage was performed using AutoDock Vina-1.5.7 software. Molecular docking simulations were used to gain insight into how these drugs bind to their targets. The binding energy between the ligand and receptor was calculated to predict affinities. A binding energy of less than 0 indicates higher affinity of binding between two molecules freely, corresponding to a smaller binding energy and a more stable binding conformation.

### Establishment of a murine DCM model

Male C57BL/6 mice (4 weeks old, weight 18–20 g) (n = 24) were purchased from the Model Animal Research Center of Nanjing University. All mice were kept in specific-pathogen-free environment (temperature 25 ± 2°C, humidity 50 ± 5%) with a 12-h light–dark cycle. Normal control mice were fed standard rodent diet and DCM model mice were fed a high-fat diet (HFD; 60% calories as fat). After four weeks of feeding, control mice were intraperitoneally injected with citrate-phosphate buffer (pH 4.2, 0.1 mmol/L). Mice in the HFD group received an intraperitoneal injection of 30 mg/kg streptozotocin (STZ; Lot No. S0130) dissolved in the same citrate-phosphate buffer for seven consecutive days. One week after the completion of STZ injections, blood glucose levels were measured. Echocardiography was performed at the end of 16 weeks. The research protocol was approved by the Animal Ethics Committee of Nanjing Medical University (license number IACUC-1903016).

### Echocardiography and statistical analysis

Mice were anesthetized with 1.5–2% isoflurane and transthoracic echocardiography was performed using a Vevo 2100 instrument (VisualSonics Inc., Toronto, Ontario, Canada). The following parameters were measured from M-mode images: left ventricular ejection fraction (EF%), left ventricular fractional shortening (FS%), early phase (E-wave), late phase (A-wave), and E/A ratio. Continuous variables in these data are expressed as means ± standard deviations, and categorical variables are expressed as frequencies with percentages. Comparisons between diabetic cardiomyopathy and non-diabetic cardiomyopathy groups were made using Welch’s *t*-test, Student’s *t*-test, or the Mann-Whitney U test, depending on whether the data met normality and chi-squaredness. All statistical analyses were conducted using R software (version 4.0.3). Two-sided P values < 0.05 were considered statistically significant.

### Reverse transcription, quantitative real-time polymerase chain reaction (qRT-PCR), and western blotting (WB)

Myocardial tissues were treated with TRIzol reagent for total RNA extraction. The RNA was reverse-transcribed into complementary DNA (cDNA) after measuring the corresponding concentration. Finally, qRT-PCR was performed to verify expression levels. The procedure for PCR is shown below: in brief, samples were incubated at 95°C for 5 min, followed by 40 cycles at 95°C for 10 s, and finally at 60°C for 20 s. 18S levels were used to normalize mRNA expression levels. The primer sequences were as follows: 5’-CTGCCGTCTGAGTGTATCGC-3′ and 5′-GCTGGGGCTGAGGAAAGTG-3 for 18S, 5’-ATGGCGTTACTGGATCTGTGC-3’ and 5’-GCGGAGAAACTGTAGTGTCCC-3’ for Psmb8; 5’-GGGGCCTGCAAAGGTTATC-3’ and 5’-TGCTGTTACGGTGCATACCC-3’ for Nfkb1; 5’-CAAGAGTGAGATCGCCCATCG-3 and 5’-TTACTTCCTGCACTAATTTGGCA-3’ for Alb; 5’-GAGCGCGTCGTACCGTATG-3 and 5’-ACTGACATCTAACTGCCTGGT-3’ for Edn1; 5’-CCCGCCTTCTACAGGTCTAAT-3’ and 5’-CTTTCTCGTTACTGCTGGACAG-3’ for Esr1.

WB was performed as follows: (i) Cells were lysed at 4°C using RIPA buffer (P0013C, Beyotime, Shanghai, China) and the cell lysates were collected. (ii) Protein concentration was measured using a BCA protein assay kit and equal amounts of proteins were separated using 10% sodium dodecyl sulfate-polyacrylamide gel electrophoresis (SDS-PAGE) (Millipore, Billerica, MA, USA) and transferred onto polyvinylidene difluoride (PVDF) membranes. (iii) The membranes were blocked with 5% skim milk for 2 h at room temperature. The membranes were cut according to the molecular weight of the target protein based on the pre-stained protein ladder. Subsequently, membranes were incubated overnight at 4°C with primary antibody (Edn1,1:1000; Psmb8,1:1000; Alb,1:1000; Nfkb1,1:1000; Esr1,1:1000; Gapdh, 1:1000). (iv) The membrane strips were incubated with the secondary antibody for 2 h at room temperature. The intensity of the protein bands was observed using Labworks software (Bio-Rad, USA) and analyzed using ImageJ software.

## Results

### Research design

**Figure 1:**
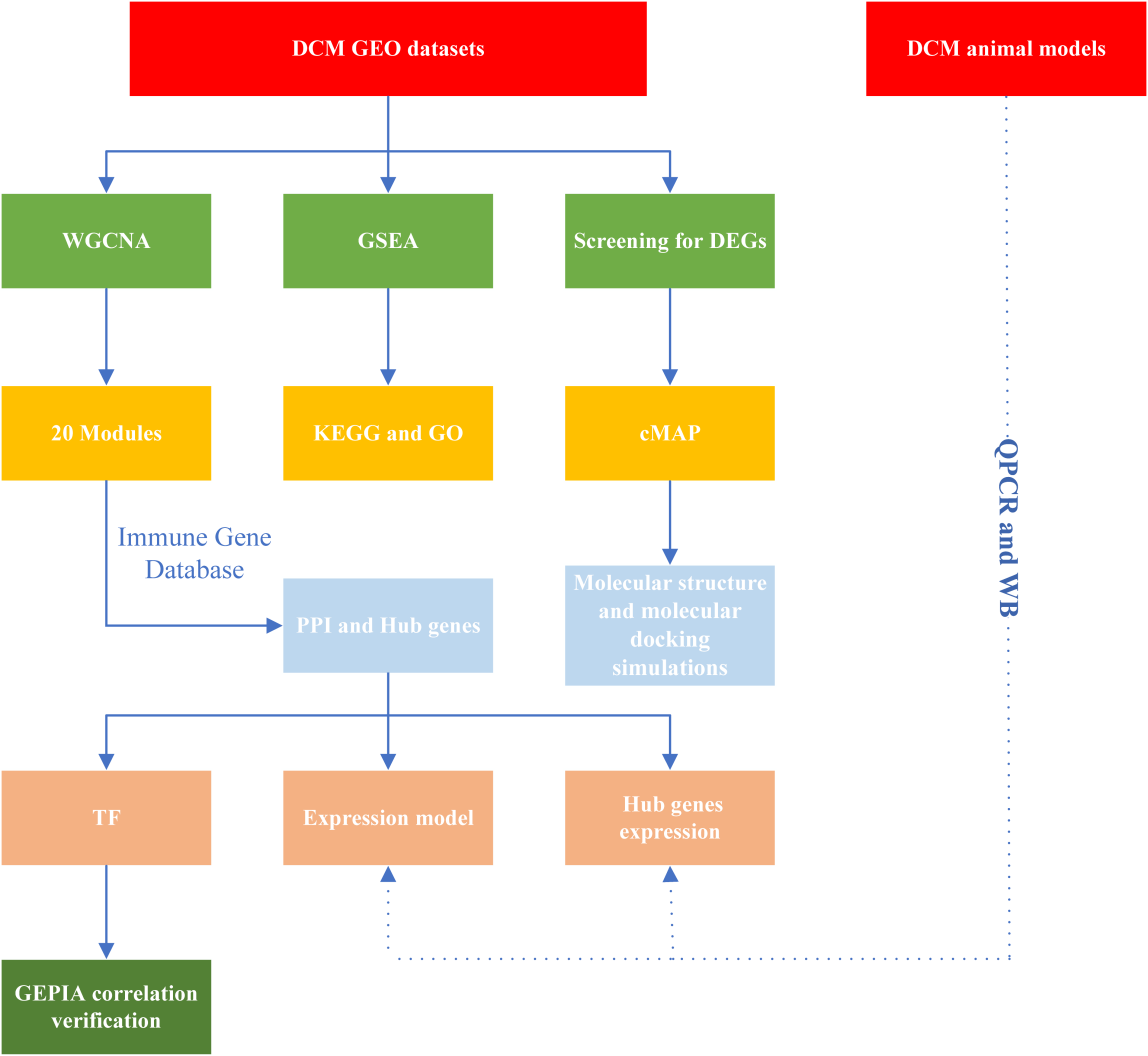
Study workflow. DCM, Diabetic cardiomyopathy; GEO, Gene Expression Omnibus; GO, Gene Ontology; PPI, protein-protein interaction; cMAP, Connectivity Map; KEGG, Kyoto Encyclopedia of Genes and Genomes; GSEA, Gene Set Enrichment Analysis; TF, transcription factors; DEGs, differentially expressed genes; WGCNA, Weight gene co-expression network analysis; GEPIA, Gene Expression Profiling Interactive Analysis; QPCR, quantitative real-time PCR; WB, Western blotting.

The flowchart is shown in **Figure 1**. In summary, hub DCM genes were identified from microarray data from the GEO database. DCM was associated with the infiltration of multiple immune cell types using the CIBERSORT test. Subsequently, WGCNA was performed to screen the core modules and identify intersections with the genes in the immune gene database. Potential drug molecules for core targets were searched as well as TFs regulating the transcription of the genes encoding them; the possibility of molecular structure docking and expression consistency was verified. Finally, qPCR was performed to verify the relationships between expression levels of the core DCM targets and clinical phenotypes in animal models.

### DCM-related immune genes

**Figure 2:**
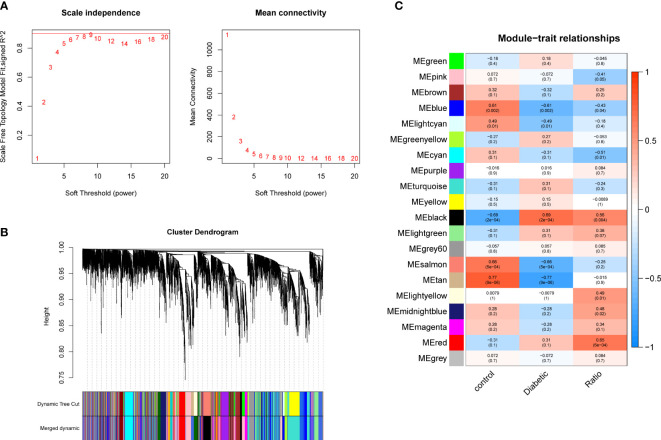
Gene co-expression modules. **(A)** Analysis of network topology for soft-thresholding powers. **(B)** Hierarchical cluster dendrogram of DCM-related genes based on one dissimilarity measure. **(C)** Module-phenotype associations.

**Figure 3:**
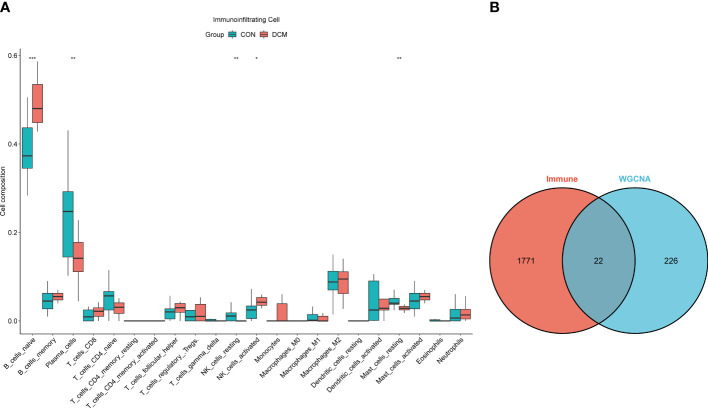
Immune infiltration analysis and intersection dataset. **(A)** Boxplot diagram of proportions of 22 types of immune cells. **(B)** Venn diagram of intersection of black modules and immune genes. * means p value <0.05; ** means p value <0.01; *** means p value <0.001.

The pickSoftThreshold parameter was calculated to set six as the optimal soft threshold. A dynamic cut tree with a merge cut height of 0.25 was used for module identification and module merging. The minimum number of genes in each network module was set to 30, producing 19 modules. The black module had the strongest correlation (correlation coefficient: 0.69, P < 0.0001) with DCM and was identified as the core module (**Figure 2**). There were significant differences in proportions of immune cells between groups and samples, with naïve B cells, plasma cells, resting NK cells, activated NK cells, and eosinophils with the most pronounced extents of DCM infiltration. The black module contained 248 genes, and the immune gene database contained 1793 genes, 22 of which were contained in both the immune database and the module (**Figure 3**).

### A PPI network for module analysis and selection of hub genes

**Figure 4:**
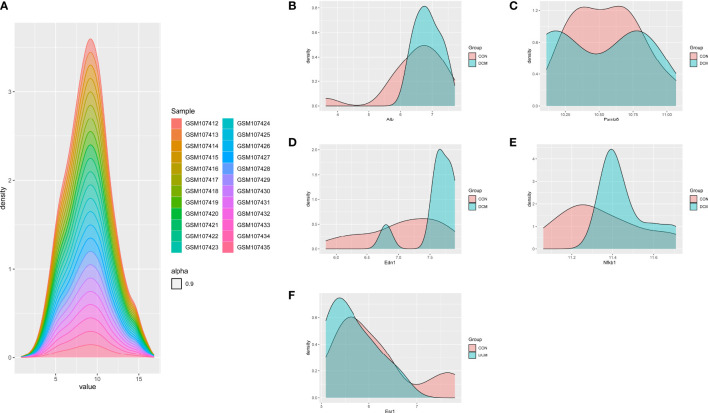
Density plots of the dataset GSE4745, showing a smooth distribution of the points along the numeric axis. The peaks of the density plot are at the locations with the highest concentration of points. **(A)** density plot of sample dataset. **(B–F)** Density plots of hub genes.

To investigate the interactions of DEGs associated with DCM occurrence and to obtain central genes, all DEGs were analyzed using STRING. Those with composite scores >0.7 suggested close associations between genes and were imported into Cytoscape for further analysis. In this network, there were 168 nodes and 527 edges; using MCODE, eight key modules were identified from the entire network (**Supplementary Figure 2**). The identified immune-related genes were further screened using cytoHubba, which produced five hub genes: *PSMB8, NFKB1, ALB, EDN1*, and *ESR1*. **Figure 4** showed the density map of the dataset GSE4745. The density plots had a smooth distribution of points along the numerical axis. The peaks of the density plots are located at locations with the highest concentrations of points. Furthermore, we show the variation of target genes in the dataset across different groups.

### GO and KEGG enrichment analysis of modules and gene sets

**Figure 5:**
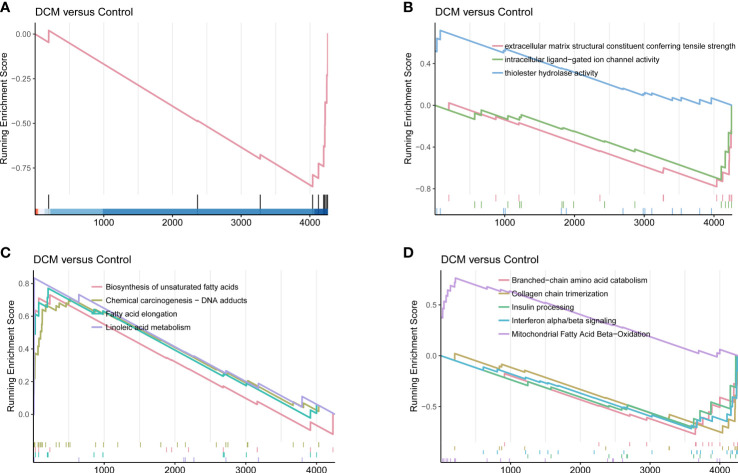
DCM-related gene set enrichment analysis. **(A)** Cell component. **(B)** Molecular function. **(C)** KEGG pathway. **(D)** Reactome.

The primary enrichments corresponding to the black module included small-molecule catabolic process, fatty acid metabolic process, ribonucleotide metabolic process, NADH metabolic process, response to hypoxia, and response to nutrient levels. Primary enrichments in the cell component (CC) included mitochondrial matrix, apical plasma membrane, cytosolic ribosome, and oxidoreductase complex. The primary enrichment in molecule function (MF) was thioester hydrolase activity. Primary enrichments in the KEGG pathway were valine, leucine, and isoleucine degradation; carbon metabolism; glutathione metabolism; and the PPAR signaling pathway (**Supplementary Figure 3**). GSEA showed that compared to control samples (screening based on |NES|>0.7,P < 0.05, and q < 0.25), the identified CC terms were complex with collagen trimers. The identified MF terms were thioester hydrolase activity, intracellular ligand-gated ion channel activity, and extracellular matrix structural constituents, conferring tensile strength. The identified reaction terms included mitochondrial fatty acid beta-oxidation, insulin processing, interferon alpha/beta signaling, collagen chain trimerization, and branched-chain amino acid catabolism. The identified pathways were linoleic acid metabolism, fatty acid elongation, unsaturated fatty acid biosynthesis, and chemical carcinogenesis (DNA adducts) (**Figure 5**).

### Murine model of DCM

A total of 24 mice were included, 12 experimental and 12 controls. There was a statistically significant difference in E/A ratios between groups (1.20 ± 0.14 vs 1.42 ± 0.22; P = 0.006), indicating significant diastolic dysfunction. Moreover, EF (65.3 ± 4.4 vs 72.3 ± 5.4; P = 0.002) and FS (35.1 ± 3.0 vs 40.8 ± 4.5; P = 0.001) values for the experimental group were significantly smaller than those of the controls, indicative of systolic dysfunction. All echocardiography parameters measured are listed in **Table 1**.

**Table 1:** Ultrasound in mice.

Group	DCM	CON	P	TOTAL
	**N = 12**	**N = 12**		**N = 24**
A, mm/s	609 ± 113	544 ± 72	0.105	577 ± 98
E, mm/s	722 ± 93	764 ± 95	0.284	743 ± 94
E/A	1.20 ± 0.14	1.42 ± 0.22	0.006	1.3 ± 0.2
Heart Rate, bpm	531 ± 78	550 ± 45	0.475	540 ± 63
Diameter;s, mm	2.34 ± 0.28	2.10 ± 0.28	0.045	2.2 ± 0.3
Diameter;d, mm	3.60 ± 0.27	3.53 ± 0.24	0.546	3.6 ± 0.25
Volume;s, ul	19.4 ± 6.1	14.9 ± 5.0	0.06	17.1 ± 5.9
Volume;d, ul	54.8 ± 10.0	52.6 ± 8.3	0.536	53.6 ± 9.1
Stroke Volume, ul	35.4 ± 4.4	37.6 ± 4.2	0.23	36.5 ± 4.4
Ejection Fraction, %	65.3 ± 4.4	72.3 ± 5.4	0.002	68.8 ± 6.0
Fractional Shortening, %	35.1 ± 3.0	40.8 ± 4.5	0.001	38.0 ± 4.7
Cardiac Output, ml/min	18.6 ± 2.7	20.6 ± 2.3	0.069	19.6 ± 2.7
LV Mass, mg	122.5 ± 12.0	134.2 ± 19.2	0.089	128.4 ± 16.7
LV Mass Cor, mg	98.0 ± 9.6	107.3 ± 15.3	0.089	102.7 ± 13.4
LVAW;s, mm	1.40 ± 0.14	1.61 ± 0.12	0.001	1.50 ± 0.16
LVAW;d, mm	0.97 ± 0.11	1.10 ± 0.09	0.004	1.03 ± 0.12
LVPW;s, mm	1.29 ± 0.12	1.40 ± 0.09	0.021	1.34 ± 0.11
LVPW;d, mm	0.90 ± 0.08	0.92 ± 0.08	0.524	0.91 ± 0.08

A, A peak; E, E peak; LV, left ventricle; LVAW, Left Ventricular Actual Weight; s, systole; d, diastole; LVPW, Left ventricular posterior wall.

### qPCR validation of hub genes and TFs

**Figure 6:**
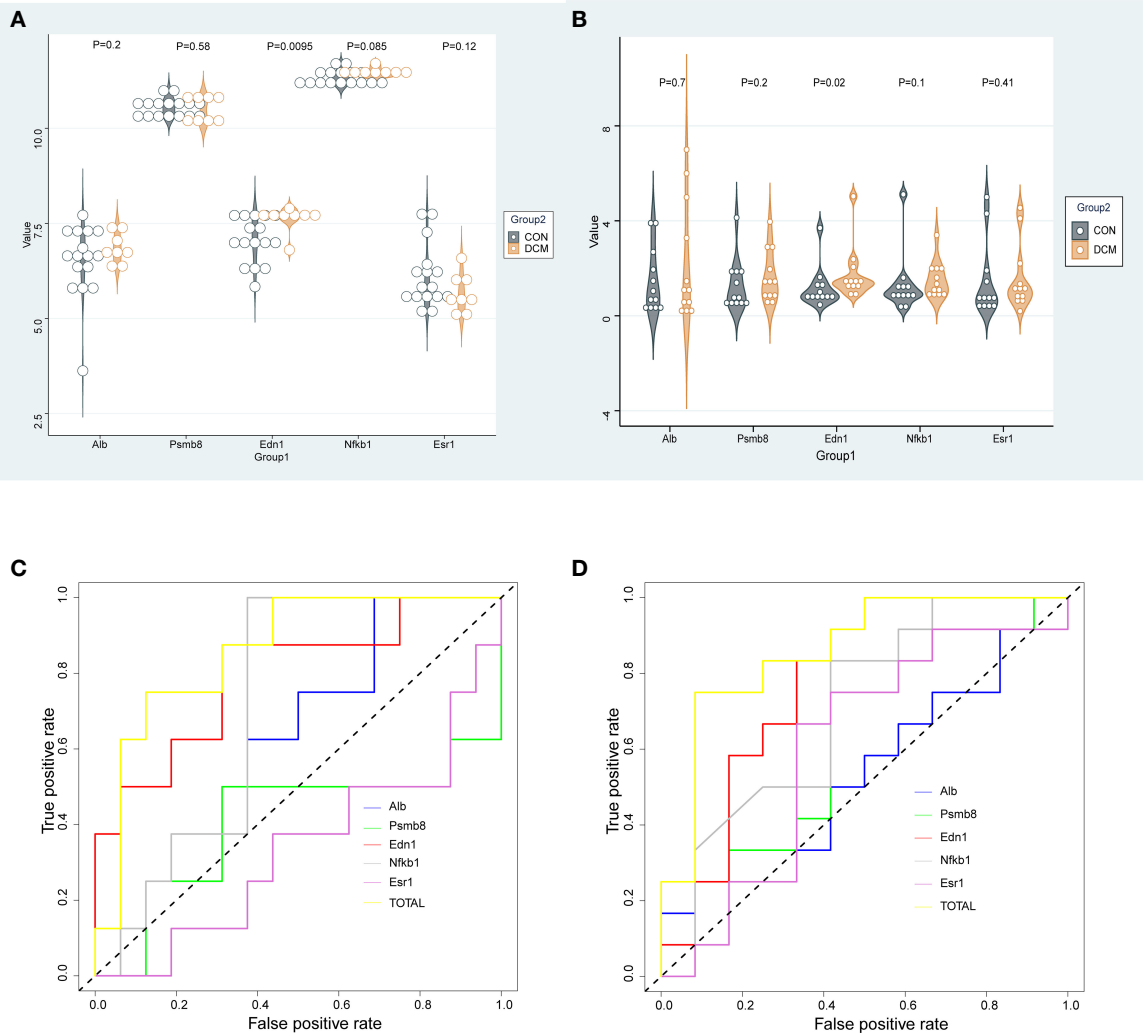
Gene expression and validation. **(A)** Dataset gene expression; **(B)** Murine DCM gene expression; **(C)** ROC for gene expression in dataset; **(D)** ROC curve for murine DCM gene expression.

**Figure 7:**
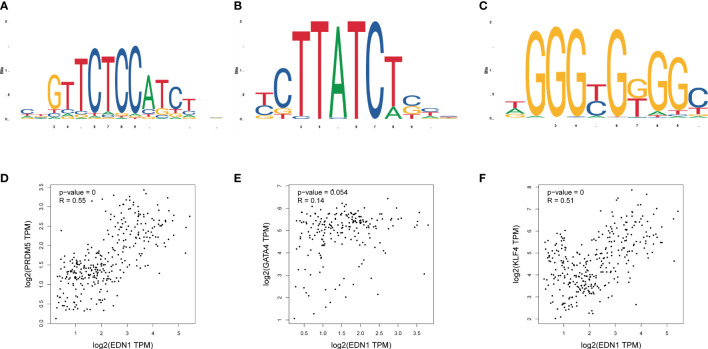
Binding sites of transcription factors and expression correlation verification. **(A)** Binding site of *Prdm5*; **(B)** Binding site of *Gata4*; **(C)** Binding site of *Klf4*; **(D)** Correlation between *Prdm5* and *Edn1* gene expression in myocardial tissue; **(E)** Correlation between *Gata4* and *Edn1* gene expression in myocardial tissue; **(F)** Correlation between *Klf4* and *Edn1* gene expression in myocardial tissue.

Relative expression levels of the hub genes are shown in **Figure 6**. The difference in gene expression between the DCM and CON groups was consistent with the PCR results. No statistically significant differences were found in the expression of four genes (*Psmb8*, *Alb*, *Nfkb1*, and *Esr1*). Only the expression of *Edn1* in the DCM group was significantly higher than that in controls (P = 0.02). The model was also built to test its predictive ability for DCM, with an area under the curve (AUC) of 0.859 for the test set and 0.861 for the validation set, showing good sensitivity and specificity. The three most likely upstream TFs (PRDM5, GATA4, and KLF4) corresponding to EDN1 were predicted by JASPAR and imported into GEPIA for validation of gene and TFr expression correlation. Among them, PRDM5 and KLF4 expression was strongly correlated with EDN1 expression, with correlation coefficients of 0.55 (P < 0.0001) and 0.51 (P < 0.0001), respectively. The TF core binding thresholds are shown in **Figure 7**.

### Validation of protein level expression

**Figure 8:**
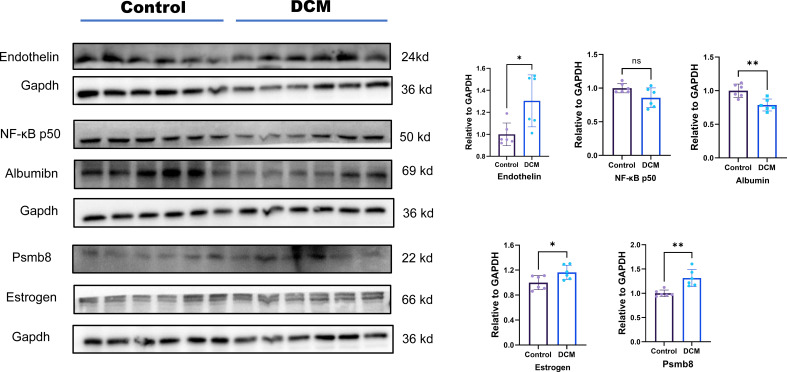
Protein level expression of hub genes. Ns means p value >0.05;* means p value <0.05; ** means p value <0.01; *** means p value <0.001.

WB was used to detect changes in the expression of target proteins. Compared with that in the normal group, the expression level of Edn1, Alb, Psmb8 and Esr1 in the DCM group was significantly changed; however, the expression levels of Nfkb1 did not differ significantly. This result is consistent with the bioinformatics prediction results and the PCR results (**Figure 8**).

### Candidate target docking and validation

**Figure 9:**
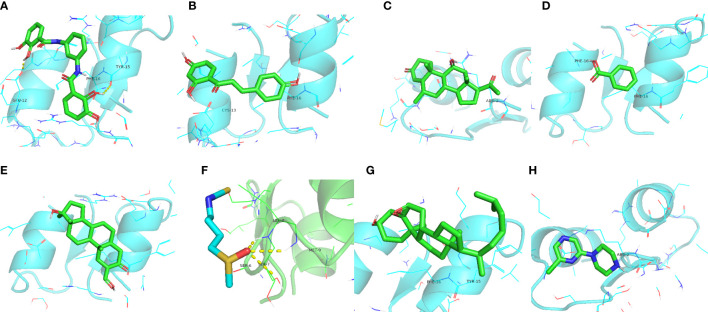
Molecular docking simulations. **(A)** EDN1 with telomerase inhibitor IX. **(B)** EDN1 with isoliquiritigenin. **(C)** EDN1 with medrysone. **(D)** EDN1 with benzoic acid. **(E)** EDN1 with oxymetholone. **(F)** EDN1 with sulforaphane. **(G)** EDN1 with epoxycholesterol. **(H)** 2-chloro-6-(1-piperazinyl)pyrazine.

Nine potential therapeutic molecules were identified from the CMap database: telomerase inhibitor IX, isoliquiritigenin, medrysone, benzoic acid, oxymetholone, sulforaphane, pevonedistat, epoxycholesterol, and 2-chloro-6-(1-piperazinyl)pyrazine (**Table 2**). Pevonedistat failed to bind to EDN1; the binding energies of the remaining candidates were -5.9, -5.3, -5.8, -3.2, -6.5, -2.3, -5.2, and -3.4 kcal/mol, respectively. The molecular docking is diagrammed in **Figure 9**.

**Table 2:** Significant small molecule chemicals.

Name	Score	Description	Target	Canonical SMILES
Telomerase Inhibitor IX	96.29	Telomerase inhibitor	TERT	C1=CC(=CC(=C1)NC(=O)C2=C(C(=CC=C2)O)O)NC(=O)C3=C(C(=CC=C3)O)O
Isoliquiritigenin	93.6	Guanylate cyclase activator	AKR1B1, HRH2, SIRT1	C1=CC(=CC=C1C=CC(=O)C2=C(C=C(C=C2)O)O)O
Medrysone	90.81	Glucocorticoid receptor agonist	NR3C1	CC1CC2C3CCC(C3(CC(C2C4(C1=CC(=O)CC4)C)O)C)C(=O)C
Benzoic acid	87.7	Protein tyrosine kinase inhibitor	ABL1, EGFR	COC(=O)C1=CC=C(C=C1)NCC2=C(C=CC(=C2)O)O
Oxymetholone	86.26	Androgen receptor agonist	AR	CC12CCC3C(C1CCC2(C)O)CCC4C3(CC(=CO)C(=O)C4)C
Sulforaphane	86.22	Antineoplastic	NFE2L2	CS(=O)CCCCN=C=S
Pevonedistat	85.24	Nedd activating enzyme inhibitor	NAE1, UBA3	C1CC2=CC=CC=C2C1NC3=C4C=CN(C4=NC=N3)C5CC(C(C5)O)COS(=O)(=O)N
Epoxycholesterol	85.16	LXR agonist	NR1H2, NR1H3	CC(C)CCCC(C)C1CCC2C1(CCC3C2CC4C5(C3(CCC(C5)O)C)O4)C
2-Chloro-6-(1-piperazinyl)pyrazine	-93.16	Serotonin receptor agonist	HTR2A,HTR2B, HTR2C	C1CN(CCN1)C2=CN=CC(=N2)Cl

Canonical SMILE: Internationally recognized atomic structure. Target: Validated targets with activity.

**Figure 10:**
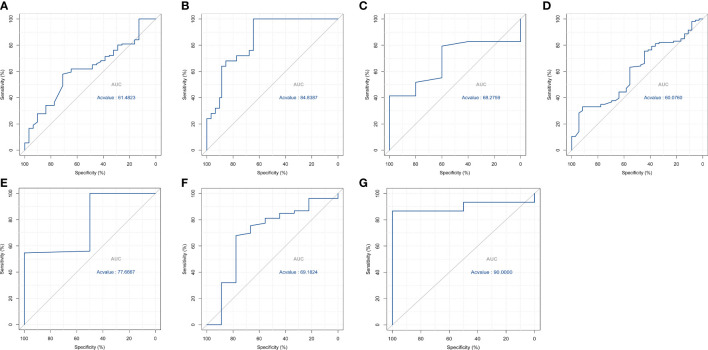
ROC curves of the virtual filter for **(A)** isoliquiritigenin; **(B)** medrysone; **(C)** benzoic acid; **(D)** oxymetholone; **(E)** sulforaphane; **(F)** pevonedistat; **(G)** 2-chloro-6-(1-piperazinyl)pyrazine.

While computational predictions usually require experimental verification, there were greater difficulties and limitations in practical implementation. Based on our experience from previous studies (12), we constructed receiver operator characteristic curve (ROC) curves corresponding to the components (active and inactive compounds) to be separated by virtual screening (**Figure 10**). Among them, medrysone, sulforaphane and 2-chloro-6-(1-piperazinyl)pyrazine were validated, corresponding to AUC of 0.848, 0.776, and 0.9, respectively.

## Discussion

DCM is characterized by a feed-forward cycle of metabolic imbalance, HF, and multiple organ dysfunction (3). Hyperglycemia and other metabolic abnormalities that induce systemic inflammation activate the immune system play roles in the progression of DCM (13). The interactions of multiple innate and adaptive immune cell populations, as well as the immune response and its regulation, also play important roles. Currently, clinical treatment strategies for DCM focus on antifibrotic agents, anti-inflammatory agents, and antioxidants, in addition to controlling metabolic disorders (14, 15). Based on immunomodulation, we expected to find key targets in diabetic cardiomyopathy to provide a highly sensitive and early method for diagnosis and targeted therapy.

A hostile environment of hyperglycemia, hyperlipidemia, hyperinsulinemia, and insulin resistance, underpinned by chronic systemic inflammation, activates the RAAS and maladaptive immune responses, changes the function of calcium, and regulates the release of cytokine in DCM (16, 17). GO function and pathway enrichment analyses showed that the products of the identified screened genes were located in the mitochondrial matrix, apical plasma membrane, cytosolic ribosome, and oxidoreductase complex; they are involved in the regulation of redox state, fatty acid metabolism, and nucleotide metabolism, which is consistent with the relevant literature. KEGG analysis showed that fatty acid synthesis and metabolism were important pathways associated with diabetic HF (18, 19). It is consistent with the reported results that diabetic cardiomyopathy is closely related to immunity (13, 20).

Through reanalysis of the GSE4745, we identified the black module as the most significant for diabetic HF with WGCNA and CIBERSORT algorithms, then identified five hub genes based on their degree values from the PPI network.: *PSMB8, NFKB1, ALB, EDN1*, and *ESR1*. Proteasome subunit beta 8) is a catalytic subunit involved in tumor infiltration and neuroinflammation (21, 22). Nuclear factor kappa B1 is a classic transcriptional regulator with multi-system involvement (23). Albumin acts as a carrier of fatty acids and a variety of enzymes, involved in the metabolism of endogenous and exogenous compounds. Hypoalbuminemia has also emerged as an independent prognosticator in many cardiovascular diseases (24, 25). *ESR1* encodes the estrogen receptor and ligand-activated TFs to regulate the estrogen signaling pathway, but its abundance in the heart is much less than that in the endometrium. It is usually relevant to the growth of galactophore tumors but has also been reported to be involved in impaired glycemic homeostasis (26). *EDN1*, located on human chromosome 6, encodes a pre-proprotein that generates a secreted peptide of the endothelin/sarafotoxin family that affects blood vessel contraction.

Identified and verified by RT-PCR in our study, the expression of *EDN1* was found to be of significantly higher level in DCM than in normal samples. A separate GWAS suggested that phosphatase and actin regulator protein 1 (PHACTR1) and EDN1 (upstream of PHACTR1) are risk factors in five vascular diseases, including CAD, migraine, cervical artery dissection, fibromuscular dysplasia, and hypertension (27). Also, binding of both mineralocorticoid and glucocorticoid receptors to the endothelin 1 EDN1 hormone response elements plays an important role in the regulation of renal sodium transport and cardiovascular physiology (28). Case-control studies have shown that PHACTR1/EDN1 is a risk factor for several vascular diseases, such as spontaneous coronary artery dissection (SCAD) (29). Some studies have suggested an association between the *EDN1* polymorphism and risk of atherosclerosis progression in great cardiac vessels and coronary arteries (30, 31). EDN1 has been implicated in the pathogenesis of microcirculation disturbances and may be critical for maintaining normal contractile function and preventing the myocardium from overstretching or even affecting blood pressure levels (32–34). Coronary microvascular dysfunction has been reported in patients with diabetes through quantitative myocardial contrast echocardiography and strain-rate imaging, as well as by adenosine stress cardiovascular magnetic resonance (CMR) (35, 36). Therefore, it is reasonable to hypothesize that EDN1 is a potential biomarker for HF induced by DCM.

We screened for potential therapeutics that can regulate the activity of TFs upstream of EDN1 to affect the pathophysiology of diabetic cardiomyopathy. Based on molecular docking, we identified telomerase inhibitor IX, isoliquiritigenin, medrysone, benzoic acid, Oxymetholone, sulforaphane, epoxycholesterol, and 2-chloro-6-(1-piperazinyl)pyrazine. In other studies, it has been shown that telomerase inhibitor IX affects tumor inhibition (37). Isoliquiritigenin has neuroprotective and antioxidant activities, while inhibiting apoptosis by ameliorating the loss of mitochondrial membrane potential and the change of nuclear morphology (38). Medrysone suppresses the effects of multiple aspects of innate and adaptive immune responses (39). Benzoic acid has been shown to improve gut functions *via* regulating enzyme activity, redox status, immunity, and microbiota (40). Oxymetholone’s anabolic properties have been studied for the treatment of damaged myocardium (41). Through the molecular target Nrf2 of Sulforaphane, it can exert a beneficial role by activating genes and molecules with antioxidant, anti-inflammatory, and anti-apoptotic properties (42). Epoxycholesterol can prevent foam cell formation in human SMCs and restore the elaboration of extracellular matrix (43). However, these molecular docking and network data require further validation by *in vivo* and *in vitro* experiments.

This study has two primary limitations. First, the verification of hub genes and their functions has only been performed in a murine model, but not in other animals or in human clinical trials. Second, experiments related to biological functions *in vivo* and *in vitro* require further study. In conclusion, in this study, we found *EDN1* to be a key gene in DCM, then predicted its regulatory TFs and identified potential therapeutic molecules. This suggests that *EDN1* has potential as a biomarker and therapeutic target for DCM.

## Data availability statement

Publicly available datasets were analyzed in this study. This data can be found here: Gene Expression Omnibus (GEO), accession number, GSE4745.

## Ethics statement

The animal study was reviewed and approved by Animal Ethics Committee of Nanjing Medical University (License number: IACUC-1903016).

## Author contributions

XL provided the general direction of the project, while QG performed data extraction, statistical analysis and writing. QZ was responsible for the establishment of animal models and the testing of model data. TZ, IC, SL, MC, XZ, and MS provided their own suggestions for revision when writing was completed. All authors have read and approved the final version of the manuscript.

## Funding

This research was supported by National Natural Science Foundation of China (grant number 2017YFC1700505).

## Acknowledgments

The authors thank the patients and investigators who participated in GEO for providing the data and Jiajin Chen, Department of Biostatistics, School of Public Health, Nanjing Medical University, for statistical guidance.

## Conflict of interest

The authors declare that the research was conducted in the absence of any commercial or financial relationships that could be construed as a potential conflict of interest.

The reviewers JS and JL declared a shared affiliation, with no collaboration, with the authors to the handling editor at the time of the review.

## Publisher’s note

All claims expressed in this article are solely those of the authors and do not necessarily represent those of their affiliated organizations, or those of the publisher, the editors and the reviewers. Any product that may be evaluated in this article, or claim that may be made by its manufacturer, is not guaranteed or endorsed by the publisher.
