# Supplementary material for: Integrated bioinformatic analysis reveals immune molecular markers and potential drugs for diabetic cardiomyopathy
Source: Front Endocrinol (Lausanne). 2022 Aug 15;13:933635. doi: 10.3389/fendo.2022.933635 (PMC9421304; doi:10.3389/fendo.2022.933635)
Supplement: Supplementary file 1 [file DataSheet_1.docx]

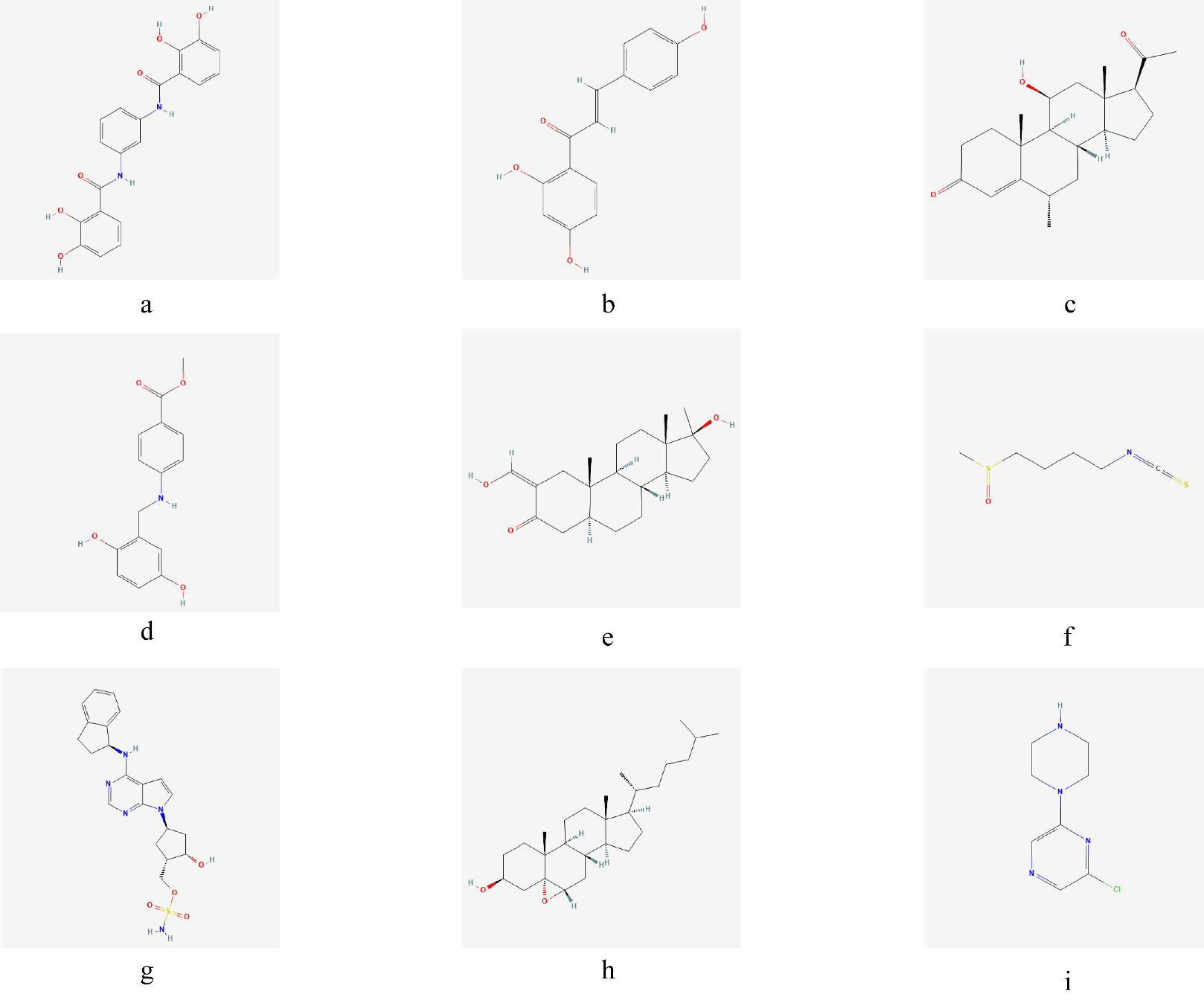


Supplementary Figure 1. Two-dimensional structures of (a) telomerase inhibitor ix; (b) isoliquiritigenin; (c) medrysone; (d) benzoic acid; (e) oxymetholone; (f) sulforaphane; (g) pevonedistat; (h) epoxycholesterol; (i) 2-chloro-6-(1-piperazinyl)pyrazine.


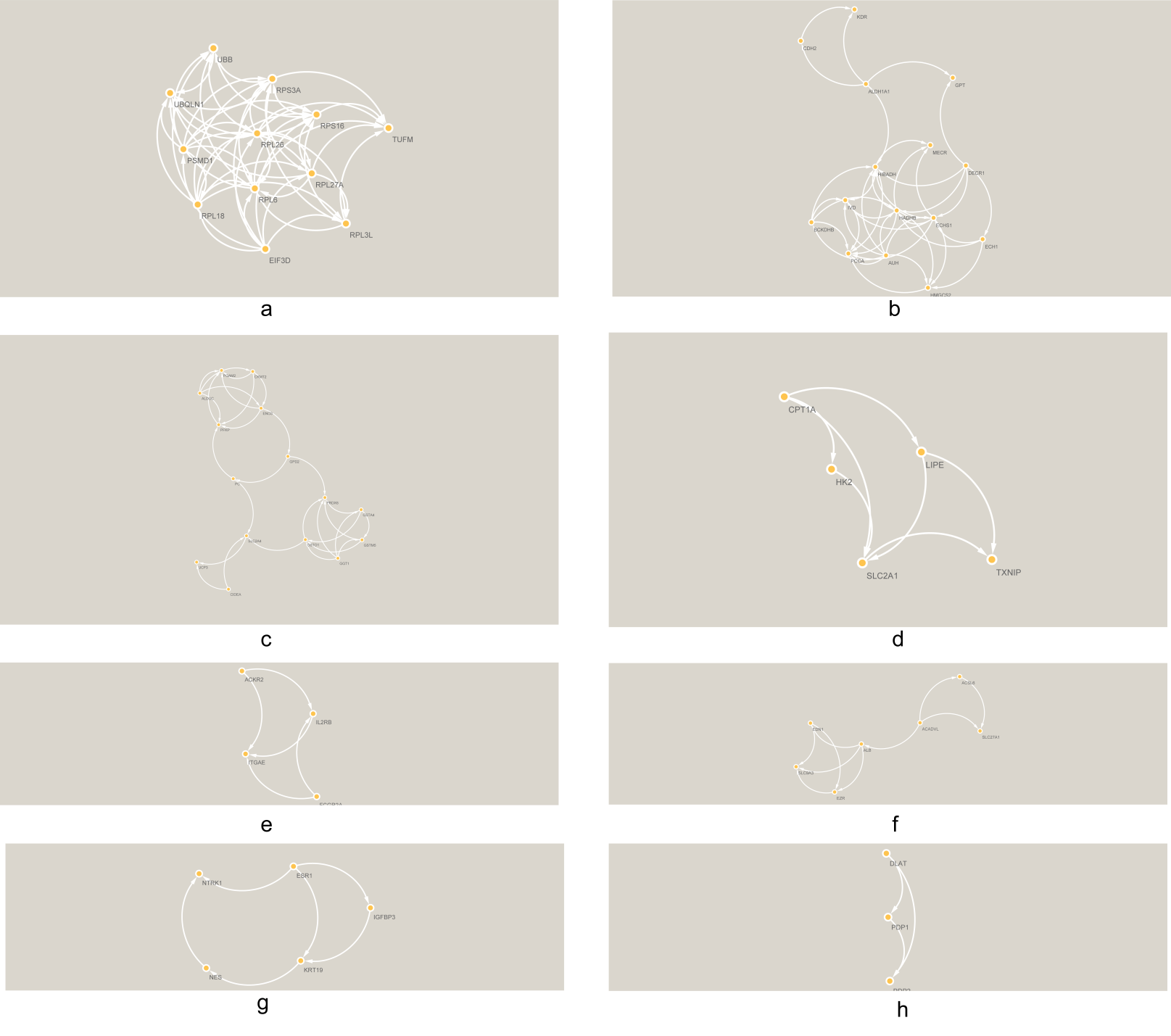


**Supplementary Figure 2**. Eight core modules in PPI network filtered using MCODE.


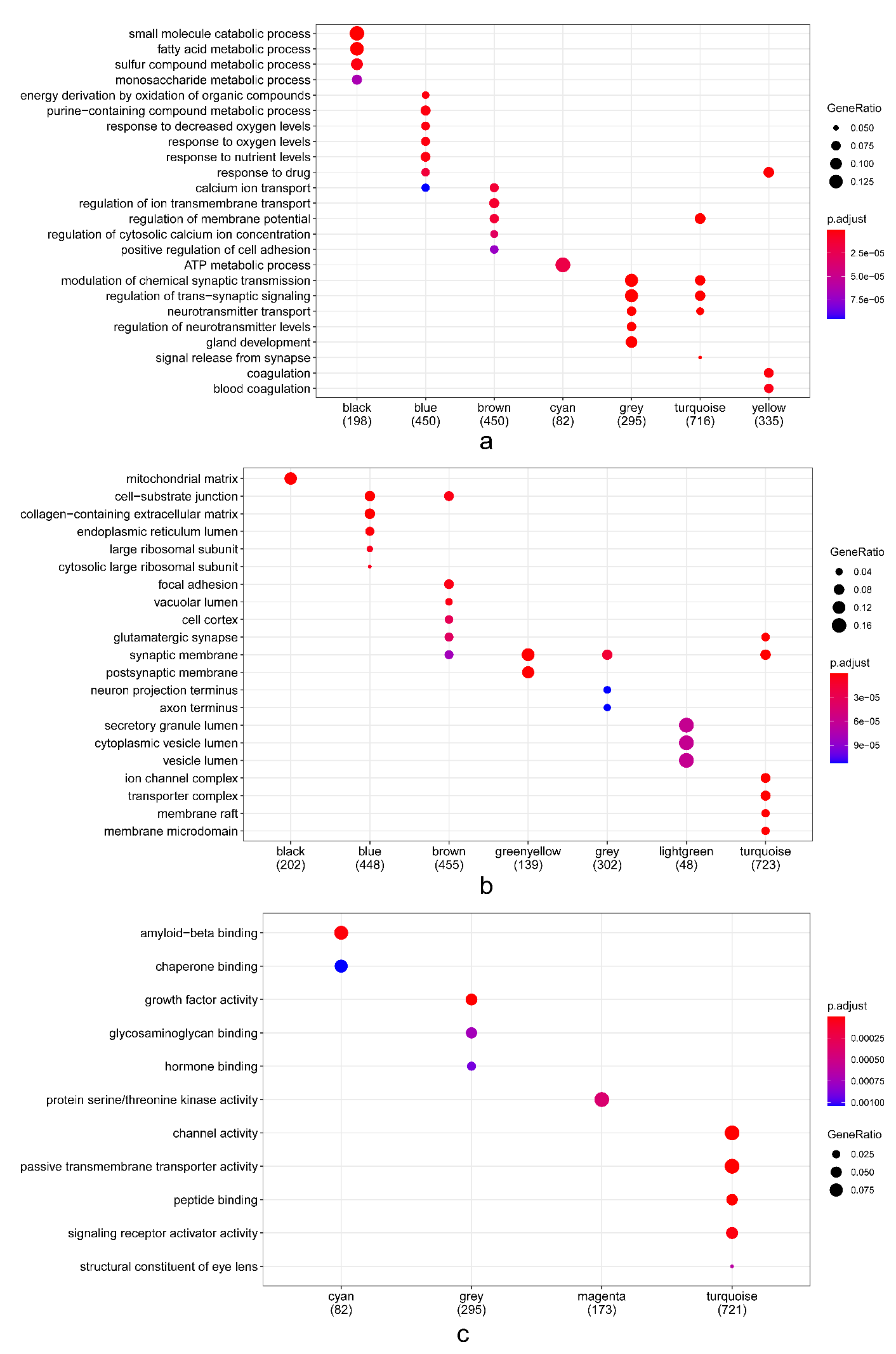


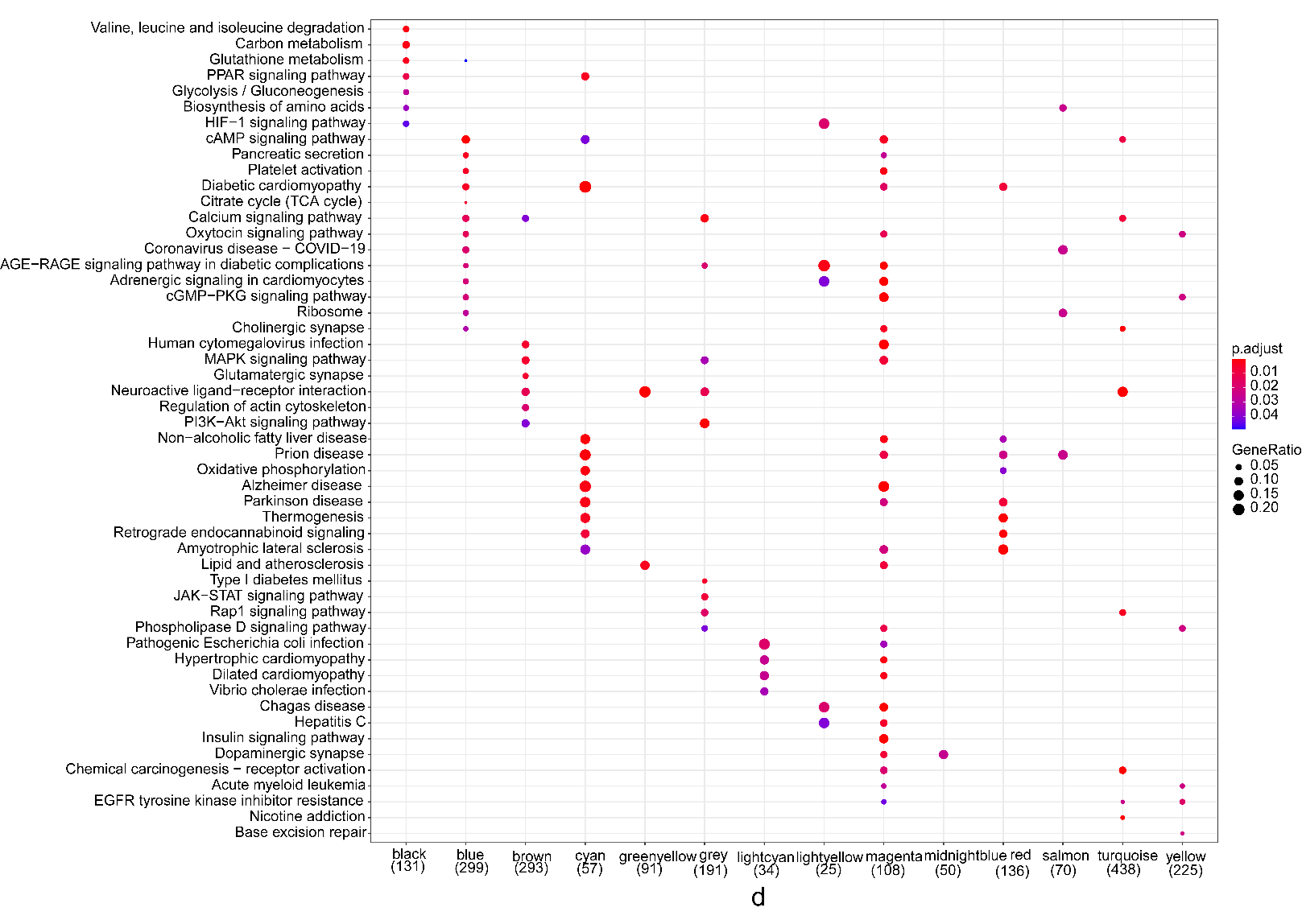


**Supplementary Figure 3**. Enrichment analyses of gene modules for (a) biological process (b) cell component; (c) molecule function; (d) KEGG pathway.
